# Towards a Robust Visual Place Recognition in Large-Scale vSLAM Scenarios Based on a Deep Distance Learning

**DOI:** 10.3390/s21010310

**Published:** 2021-01-05

**Authors:** Liang Chen, Sheng Jin, Zhoujun Xia

**Affiliations:** School of Mechanical and Electric Engineering, Soochow University, Suzhou 215131, China; jsdd25@163.com (S.J.); xzj10176719@163.com (Z.X.)

**Keywords:** visual place recognition, vSLAM, deep distance learning, multi-constraint loss, CNN

## Abstract

The application of deep learning is blooming in the field of visual place recognition, which plays a critical role in visual Simultaneous Localization and Mapping (vSLAM) applications. The use of convolutional neural networks (CNNs) achieve better performance than handcrafted feature descriptors. However, visual place recognition is still a challenging task due to two major problems, i.e., perceptual aliasing and perceptual variability. Therefore, designing a customized distance learning method to express the intrinsic distance constraints in the large-scale vSLAM scenarios is of great importance. Traditional deep distance learning methods usually use the triplet loss which requires the mining of anchor images. This may, however, result in very tedious inefficient training and anomalous distance relationships. In this paper, a novel deep distance learning framework for visual place recognition is proposed. Through in-depth analysis of the multiple constraints of the distance relationship in the visual place recognition problem, the multi-constraint loss function is proposed to optimize the distance constraint relationships in the Euclidean space. The new framework can support any kind of CNN such as AlexNet, VGGNet and other user-defined networks to extract more distinguishing features. We have compared the results with the traditional deep distance learning method, and the results show that the proposed method can improve the performance by 19–28%. Additionally, compared to some contemporary visual place recognition techniques, the proposed method can improve the performance by 40%/36% and 27%/24% in average on VGGNet/AlexNet using the New College and the TUM datasets, respectively. It’s verified the method is capable to handle appearance changes in complex environments.

## 1. Introduction

Visual place recognition is a critical and challenging problem in visual Simultaneous Localization and Mapping (vSLAM) applications. Given a query image, the purpose of visual place recognition is to find the most similar images upon repeated traversals, which is also known as loop closure detection [1]. Visual place recognition is especially important for vSLAM to perform loop closure detection to eliminate accumulated errors. Additionally, a robust tracking module is necessary for accurate pose and map in vSLAM systems. However, in practical use, tracking failure is inevitable due to reasons such as fast motion, blurred images, excessive changes in camera’s angle of view, lack of texture, etc. Therefore, an efficient relocalization module is indispensable. In modern feature-based vSLAM systems, such as ORB-SLAM [2], there are two main components to relocalize the robot. The first step is searching candidate keyframes (visual place recognition) and the second step is keypoint feature matching (metric localization). Without accurate visual place recognition, trajectory drift will occur and an ambiguous map of unknown environment will be constructed in large-scale localization and mapping [3,4]. However, visual place recognition remains a challenge problem because of perceptual aliasing and perceptual variability problems. Perceptual aliasing, also called false positive, is the case that images from different places look similar and treated as from the same place. Perceptual variability, also called false negative, is the case that images from the same place look different and are recognized as from different places by mistake due to significant appearance variations, such as seasonal variation, viewpoint variation, illumination variation, dynamic objects and so on [5]. Moreover, for real-time autonomous robotics, time performance is of great significance to be considered at deployment. Therefore, in large-scale vSLAM scenarios, with the continuous increasing of map, visual place recognition needs to still maintain efficiency.

Traditional handcrafted feature descriptors are frequently used for feature extraction in visual place recognition. The scale-invariant feature transform (SIFT) feature descriptor is a widely used image feature algorithm, which has certain invariance to scale, rotation and illumination [6]. The problem with the SIFT method is that it requires a large amount of calculations in feature extraction. The speeded-up robust feature (SURF) feature descriptor [7], oriented FAST and rotated BRIEF (ORB) feature descriptor [8] and histogram of oriented gradient (HOG) feature descriptor [9] all improve the efficiency at the expense of performance. These handcrafted feature descriptors are usually integrated into a Bag of Visual Words (BoVW) model, which clusters a large number of feature descriptors offline and finally forms a visual vocabulary to represent images [10]. In order to further improve real-time performance, Gálvez-López et al. [11] proposed to establish a vocabulary tree to discretize the binary description space, which makes vocabulary query more efficient. However, traditional handcrafted feature descriptors cannot cope with strong environmental appearance changes.

Recently, the use of deep learning technology in visual place recognition has obtained better performance than those handcrafted methods because deep neural networks can extract more comprehensive images features [12,13,14]. Deep learning can extract abstract and high-level features of the input image through multi-layer networks, which is more robust to appearance changes [15,16]. Sunderhauf et al. [17] used AlexNet [13] trained on the ImageNet dataset [18] to extract features. Xia et al. [19] proposed to use PCANet to extract features as image descriptors. The method in [20] used AMOSNet and HybridNet, which were trained on a large-scale scene classification dataset. Sun et al. [21] proposed a point-cloud-based place recognition task using CNN models. Camara et al. [22] proposed a two-stage visual place recognition system, which employs the activations of different layers of VGGNet [23] to encode images. The above methods are superior due to the features can be automatically learned by deep learning models. However, the neural networks used in the previously mentioned methods were designed for image classification tasks but not specifically developed for the characteristics of visual place recognition tasks. Features suitable for image classification tasks are not necessarily suitable for visual place recognition tasks, because the models used to extract these features are not designed to deal with strong environmental appearance changes that commonly occur in vSLAM tasks. In this paper, visual place recognition is better performed by discriminatively training a network to embed images in the Euclidean space where small Euclidean distances represent similar places, as opposed to using handcrafted feature descriptors or feature vectors extracted from generic deep learning networks.

Recently, some research works significantly improved recognition results by focusing on extracting features from salient regions and discarding confusing regions. Tolias et al. [24] proposed a method named Regional Maximum Activation of Convolutions (R-MAC), which used max-pooling operation to encode image regions. Khaliq et al. [25] used a lightweight CNN to detect local features and combined them with Vector of Locally Aggregated Descriptors (VLAD) [26] encoding method. These methods significantly improved the robustness of visual place recognition. However, they may not be fast enough for large-scale real-time vSLAM due to the slow local feature extraction. On the one hand, it is time-consuming for most existing algorithms to produce salient regions from a single image. For example, the Edge Boxes [27] algorithm takes nearly 1.8 s to process an image on a standard desktop machine [28]. On the other hand, to guarantee the robustness of visual place recognition, it is necessary to increase the number of salient regions, which is more complicated than simply using global features since each region requires a CNN forward propagation to extract features. In contrast to these methods, once the CNN network is well-trained in this paper, it can be used to extract more distinguishing global features with a single CNN forward propagation.

Instead of focus on how to extract more comprehensive features from images, some research address the visual place recognition problem by matching sequences of images [29,30,31,32]. SeqSLAM [29] is a visual place recognition technique using confusion matrix by subtracting patch-normalized sequences of images to find the matched place, which shows robust to seasonal and illumination variations. Oishi et al. [30] proposed SeqSLAM++, which generalizes the SeqSLAM to deal with image deformations and large view direction changes. However, the calculation of these methods is very time-consuming, especially for large-scale place recognition tasks. In contrast to these methods, sequence search techniques are not applied in this paper. Visual place recognition is performed by pure image retrieval based on the Euclidean distance of the extracted feature vectors.

Deep distance learning is of great significance in learning visual similarity. Recently, a specially designed triplet loss combined with CNN feature extraction has achieved good performance in face recognition [33], person re-identification [34,35], camera-LiDAR place recognition [36] and radar place recognition [37,38,39] tasks. The main concept behind the triplet loss is to minimize the distances of the same category images and maximize those of other categories in the Euclidean space. Inspired by these work, this research focuses on learning powerful global features to improve the performance and robustness of visual place recognition under strong appearance changes. In this paper, we develop our approach based on a novel framework. The basic idea of this framework is to minimize the distances of images from the same place and maximize those of images from different places. Therefore, a so-called multi-constraint loss function is customized for the task of visual place recognition to learn more distinguishing image representation. Consequently, the features extracted from our proposed method are not only robust to significant appearance changes, but also fast enough for inference in large-scale visual place recognition applications. The experimental results on several mainstream datasets indicate that our method can achieve promising results and outperforms several off-the-shelf approaches.

## 2. Framework and Methods

**Figure 1:**
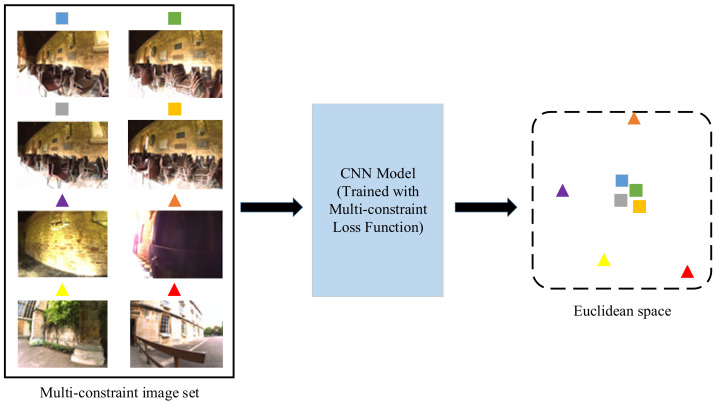
The framework of the proposed multi-constraint deep distance learning.

The framework of our proposed multi-constraint deep distance learning for visual place recognition problem is shown in Figure 1. In this paper, we use the CNN model trained by a novel deep distance learning method to extract feature vectors from images and compute similarity by comparing the Euclidean distance of two feature vectors. Our main idea is to improve the feature extraction ability of the CNNs based on a novel multi-constraint loss function that can represent the essential distance relationship. The kernel is to extract more distinguishing feature descriptors to better learn the distance constraints in the Euclidean space for the visual place recognition tasks. The framework can support any kind of CNN such as the well-known AlexNet [13], VGGNet [23] and other user-defined networks. In this work, we adopt AlexNet and VGGNet as two instances to illustrate multi-constraint deep distance learning.

As shown in Figure 1, we construct multi-constraint image sets to train the CNN model based on the multi-constraint loss function to obtain more effective feature representations in the Euclidean space that the images from the same place are closer to each other. Once the CNN model is well-trained, it can be used for visual place recognition. Instead of inputting a single frame into the neural network, consecutive multiple frames are combined as input to derive their low-dimensional feature vectors, of which the Euclidean distance can be directly used to metric the similarity of different places. If the Euclidean distance between the images being compared is lower than a given threshold τ, a place recognition hypothesis can be proposed. The threshold τ may be preset manually. In this paper, we choose τ which maximizes the recall rate with perfect precision.

### 2.1. CNN Based Image Feature Extraction

Deep learning related research have shown that CNNs have strong ability in feature extraction, especially for images. Fully Connected layers in CNNs are used to integrate high level features and often encode diverse visual features. Therefore, these fully connected layers can be used as feature extractors to derive the feature vectors.

In the instance retrieval task that is similar to the visual place recognition task, the image feature extracted from different layers exhibit different performances [40]. Experiments show that the generalization ability of the top layers is weaker than that of lower layers. For example, experimental results on AlexNet show that FC6, FC7 and FC8 are in descending order of retrieval accuracy [40,41]. What’s more, the intermediate layers feature of AlexNet and VGGNet outperform the fully connected layers feature in tasks of image search and classification [40,42]. It is noted that, in our research, the architecture of AlexNet and VGGNet is simplified for the visual place recognition problem, i.e., the original fully connected layers of AlexNet and VGGNet are replaced with a customized fully connected layer with adjustable dimensions. Notice that this fully connected layer is taken as the feature extractor to encode the feature representation learned from deep distance learning method with adjustable vector lengths.

### 2.2. Triplet Loss

In a standard triplet loss method, three images are combined into a tuple. Then, a batch of tuples are taken as inputs. Suppose i,j,k are the sequence numbers of the images selected from the image set, if the following relationships are met, they can be a suitable tuple:

- Image *i* and image *j* are not the same image and from the same category.
- Image *i* and image *k* are from different categories.

Consider a tuple denoted as $\left(x^{a},x^{p},x^{n}\right)$, where *x^a^* and *x^p^* belong to the same category while *x^n^* and *x^a^* come from the different categories. The purpose of the triplet loss function is to optimize the CNNs to learn the representation *f*(*x*) of image *x*. The ideal image representation of a tuple input $\left(x^{a},x^{p},x^{n}\right)$ should meet the following distance relationship:

(1) $${\left\|f(x^{a})-f(x^{p})\right\|}_{2}^{2}+\alpha \leq {\left\|f(x^{a})-f(x^{n})\right\|}_{2}^{2}$$

where α is a value of the margin between image pairs of same category and different categories. Moreover, all feature vectors are constrained in the hypersphere ${\left\|f(x)\right\|}_{2}^{}=1$ to avoid the loss exceeding zero easily [34]. Formally, the triplet loss function is defined as:

(2) $$L_{tri}(x^{a},x^{p},x^{n})=\max\left\{{\left\|f(x^{a})-f(x^{p})\right\|}_{2}^{2}+\alpha -{\left\|f(x^{a})-f(x^{n})\right\|}_{2}^{2},0\right\}$$

The training purpose is to learn a distinguishing image representation, where the distance between *f*(*x^a^*) and *f*(*x^p^*) is minimized and the distance between *f*(*x^a^*) and *f*(*x^n^*) is maximized.

**Figure 2:**
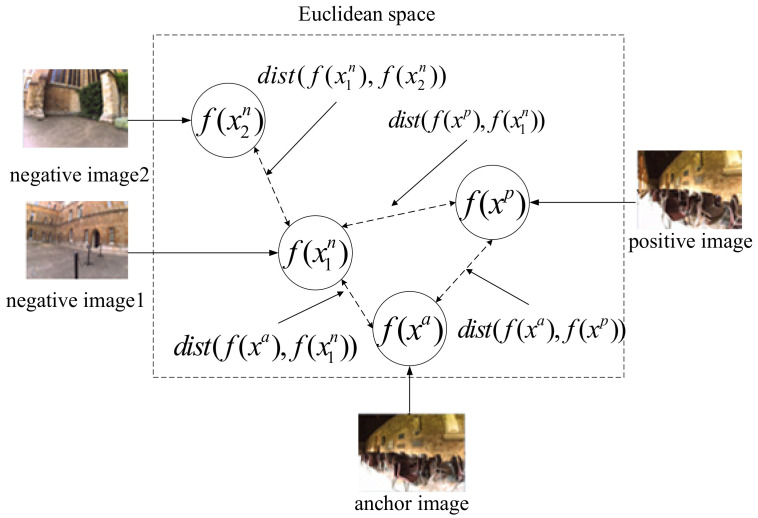
The possibly anomalous distance relationship caused by the triplet loss.

The use of triplet loss has been successful in certain research areas such as face recognition and person re-identification. Concerning place recognition problem, our previous work [43] proposed a multi-tuplet clusters loss (an improved triplet loss) that is customized for distance learning of visual loop closure detection. The method is more competitive than the state-of-art approaches in complex environments with strong appearance changes. However, there are still some drawbacks rooted in the triplet loss should be further improved. In order to maximize the distance between the matched and the mismatched image pairs for each tuple, it might lead to anomalous distance relationships as shown in Figure 2. We define a function *dist*(), which represents Euclidean distance between feature vectors. To optimize this tuple, the operation of maximizing $dist(f(x^{a}),f(x_{1}^{n}))$ might lead to an undesirable result of minimizing $dist(f(x_{1}^{n}),f(x_{2}^{n}))$, while negative image1 and negative image2 may come from different places. 

Therefore, it’s necessary to develop a novel metric to learn the multiple constrained distance relationships in the visual place recognition, thus the so-called multi-constraint loss is proposed.

### 2.3. Multi-Constraint Loss

The triplet loss only constrains two aspects of distance relationships, i.e., the distance constraint between *f*(*x^a^*) and *f*(*x^p^*) and the constraint between *f*(*x^a^*) and *f*(*x^n^*). To overcome the weakness of triplet loss, we proposed a novel loss function named multi-constraint loss, which is dedicated to describe the intrinsic distance constraints in the problem of visual place recognition.

**Figure 3:**
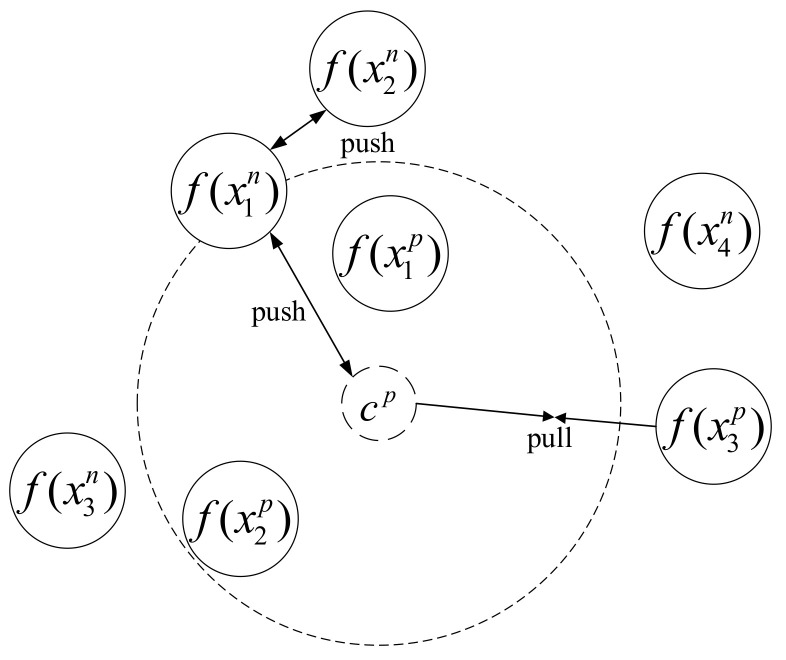
Illustration the idea of multi-constraint loss.

Different from triplet loss, we construct multi-constraint image set $X=(X^{p},X^{n})$ rather than tuple as the input. Each multi-constraint image set contains two different image sets: one positive image set and one negative image set. The former one $X^{p}=(x_{1}^{p},\ldots ,x_{A}^{p})$ contains *A* images from the same place and the latter set $X^{n}=(x_{1}^{n},\ldots ,x_{B}^{n})$ contains *B* images from different places. It is assumed that images from the same place should be close to each other and images from different places should be separated far apart in the feature space. The principle of the multi-constraint loss is illustrated in Figure 3, where $c^{p}$ denotes the center point of images in the positive image set, and it can be computed as:

(3) $$c^{p}=\frac{1}{A}\sum_{i=1}^{A}f(x_{i}^{p})$$

Different from the triplet loss, the multi-constraint loss constrains three aspects of distance relationships. The first constraint is about the distance relationship between the positive image and the center point, which is called distance relationship 1 (for short DR1). The second constraint is about the distance relationship between the negative image and the center point, which is called distance relationship 2 (for short DR2). The third constraint is about the distance relationship between images in the negative image set, which is called distance relationship 3 (for short DR3).

For a multi-constraint image set $X=(X^{p},X^{n})$, the desired distance relationships of DR1 and DR2 should satisfy the following condition:

(4) $${\left\|f(x_{i}^{p})-c^{p}\right\|}_{2}^{2}+\alpha \leq {\left\|f(x_{j}^{n})-c^{p}\right\|}_{2}^{2},\forall 1\leq i\leq A\;and\;1\leq j\leq B$$

where α is the predefined hyperparameter.

Meanwhile, in order to tackle the problem existing in triplet loss as shown in Figure 2 (i.e., to maximize $dist(f(x_{j}^{n}),f(x_{k}^{n}))$, where image $x_{j}^{n}$ and image $x_{k}^{n}$ are negative images but from different places. According to the distance relationship of the triangle, we have to meet the following constraint between DR1 and DR3:

(5) $${\left\|f(x_{i}^{p})-c^{p}\right\|}_{2}^{2}+\beta \leq {\left\|f(x_{j}^{n})-f(x_{*}^{n})\right\|}_{2}^{2},\forall 1\leq i\leq A\;and\;1\leq j\leq B$$

where β is the predefined hyperparameter, and $x_{*}^{n}$ can be randomly selected from the negative image set with $x_{*}^{n}\neq x_{j}^{n}$. As mentioned before, there is only a single image from each place in $X^{n}$. Therefore, $x_{*}^{n}$ and $x_{j}^{n}$ are from different places. In a multi-constraint image set, the distance constraints can be converted to the minimization problem of the following objective,

(6) $$\begin{array}{l} L_{multi}(X^{p},X^{n})=\max\{0,{\left\|f(x_{i}^{p})-c^{p}\right\|}_{2}^{2}+\alpha -{\left\|f(x_{j}^{n})-c^{p}\right\|}_{2}^{2}\}\\ +\max\{0,{\left\|f(x_{i}^{p})-c^{p}\right\|}_{2}^{2}+\beta -{\left\|f(x_{j}^{n})-f(x_{*}^{n})\right\|}_{2}^{2}\},\;\forall 1\leq i\leq A\;and\;1\leq j\leq B \end{array}$$

If the multi-constraint image set is large, the optimization process would be too time-consuming to be carried out in an embedded system. To accelerate this process, we can first find out the farthest positive image $x_{farthest}^{p}$ from $c^{p}$ by $\underset{i}{\max}({\left\|f(x_{i}^{p})-c^{p}\right\|}_{2}^{2}),i=1,2,\ldots ,A$, and the nearest negative image $x_{nearest}^{n}$ from the $c^{p}$ by $\underset{j}{\min}({\left\|f(x_{j}^{n})-c^{p}\right\|}_{2}^{2}),j=1,2,\ldots ,B$. Thus, the loss function can be simplified as:

(7) $$\begin{array}{l} L_{multi}(X^{p},X^{n})=\max\{0,{\left\|f(x_{farthest}^{p})-c^{p}\right\|}_{2}^{2}+\alpha -{\left\|f(x_{nearest}^{n})-c^{p}\right\|}_{2}^{2}\}\\ +\max\{0,{\left\|f(x_{farthest}^{p})-c^{p}\right\|}_{2}^{2}+\beta -{\left\|f(x_{nearest}^{n})-f(x_{*}^{n})\right\|}_{2}^{2}\} \end{array}$$

Therefore, multi-constraint loss can learn effective feature representations that can satisfy the relative distance relationship in visual place recognition, and can make the extracted features more discriminative. Instead of selecting anchor image, the center point *c^p^* is used to constrain the overall distance relationship between positive images and negative images. Moreover, the construction of the multi-constraint image set guarantees all images from the same place will move closer to the center point *c^p^*. Thus, the overall distance relationship between positive images and negative images can be measured and constrained by the center point *c^p^* derived by the clustering method. In the original tuple generation approach, the training process is inefficient, which may cause computational burden for the deployment use of visual place recognition in real-time autonomous robotics. Therefore, the idea of multi-constraint image set is introduced, which can guarantee faster training and testing process based on ‘image place’. It facilitates the proposed method to handle consecutive frames, which can speed up the testing process. 

### 2.4. Construction of Multi-Constraint Image Set

Existing datasets for visual place recognition task usually contain images and ground-truth labels. We mark images from the same place with the same label. For the *k*th place that contains *u* images, if the distance relationship in Equation (8) is met, we add the image *x_i_* into the temporary positive image set $X_{tem}^{p}$:

(8) $${\left\|f(x_{i}^{})-c_{}^{p}\right\|}_{2}^{2}>\gamma ,\;\forall 1\leq i\leq u$$

For other places, e.g., the *t*th place, if the distance relationship in Equation (9) is met, we add the image *x_j_* into the temporary negative image set $X_{tem}^{n}$:

(9) $${\left\|f(x_{j}^{})-c_{}^{p}\right\|}_{2}^{2}<\gamma^{\prime },\;\forall 1\leq j\leq v$$

where *v* is the total number of images in the *t*th place. Thus, we can construct the suitable multi-constraint image set by Algorithm 1. Each image in the training set has a corresponding place label. The number of images contained in each place is not fixed. Some places contain only one image and some other places contain multiple images. At first, we traverse each image and calculate the center point of the place to which this image belongs. Then, a temporary positive sample set $X_{tem}^{p}$ and a temporary negative sample set $X_{tem}^{n}$ are constructed according to the distance relationship. However, the lengths of $X_{tem}^{p}$ and $X_{tem}^{n}$ do not necessarily match the input lengths defined by $X^{p}$ and $X^{n}$. Hence, we can adjust the length of $X_{tem}^{p}$ and $X_{tem}^{n}$ as *A* and *B*, respectively.
**Algorithm 1.** Method to construct multi-constraint image sets.Input: Training set with place labels $\left\{\left(x_{i},y_{i}\right)\right\},i=1,2,\ldots ,n$; Output: Multi-constraint image sets X; 1: Extract the feature vector for each training image; 2: for each *i* in {1, 2, ……, *n*} do 3:  Find *u* images from the same place with *x_i_*; 4:  Calculate the center point *c^p^*; 5:  for each *j* in {1, 2, ……, *n*} and *j* ≠ *i* do 6:    Find images from the same place & satisfy the distance relationship ${\left\|f(x_{j}^{p})-c_{}^{p}\right\|}_{2}^{2}>\gamma$. Add these images into positive image set $X_{tem}^{p}$; 7:    Find images from different places & satisfy the distance relationship ${\left\|f(x_{j}^{})-c_{}^{p}\right\|}_{2}^{2}<\gamma^{\prime }$. Add these images into negative image set $X_{tem}^{n}$; 8:  end for 9:  if $len(X_{tem}^{n})>B$ then 10:    Randomly select *B* images from $X_{tem}^{n}$; 11:  end if 12:  if $0<len(X_{tem}^{n})<B$ or ($len(X_{tem}^{n})=0$ and $len(X_{tem}^{p})\neq 0$) then 13:    Randomly select $\left(B-len(X_{tem}^{n})\right)$ images from different places. 14:  end if 15:  if $len(X_{tem}^{p})>A$ then 16:    Randomly select *A* images from $X_{tem}^{p}$; 17:  end if 18:  if $0<len(X_{tem}^{p})<A$ or ($len(X_{tem}^{p})=0$ and $len(X_{tem}^{n})\neq 0$) then 19:    Randomly copy $\left(A-len(X_{tem}^{p})\right)$ images from the same place. 20:  end if 21:  if $X^{p}$ and $X^{n}$ exist then 22:    Add $\left(X^{p},X^{n}\right)$ into X; 23:  end if 24: end for 25: return X

### 2.5. Training Process

**Figure 4:**
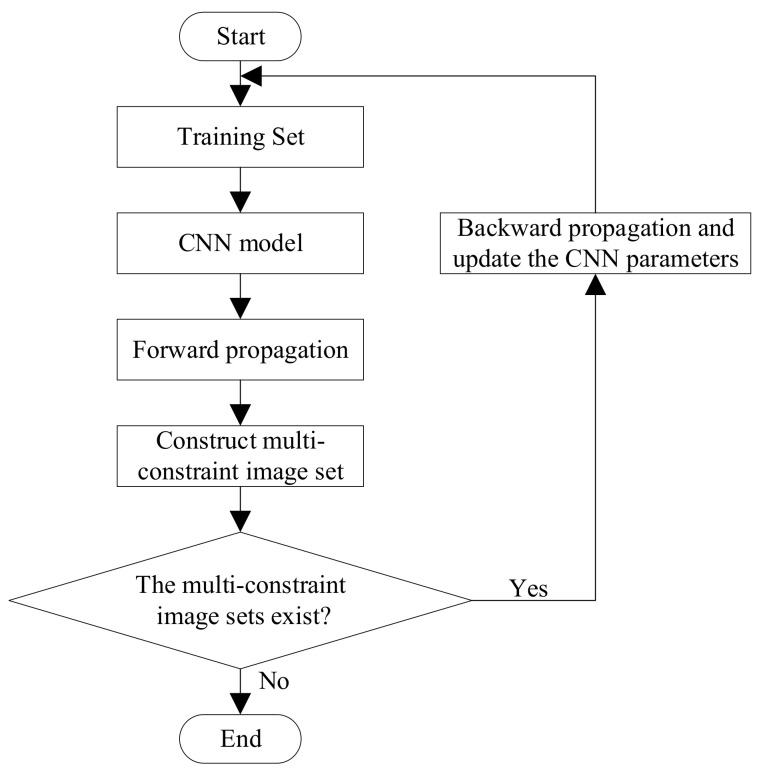
The diagram of the training process.

The diagram of the training process is shown in Figure 4. First, we divide the training set into several mini-batches with the size of (*A* + *B*). These mini-batches are input into CNN models which share parameters updated by the multi-constraint loss function. In this way, feature vector of each image can be derived by forward propagation. Then, we can construct suitable multi-constraint image sets according to the Algorithm 1. If there exist multi-constraint image sets, we will update the parameters of the CNN model using the multi-constraint loss function. If there isn’t any multi-constraint image set existing, the training process ends and it means the CNN is successfully trained.

In this work, the sizes of the positive image set and the negative image set are set to be 4 and 4, respectively. The batch size is set to be 5. The hyperparameters *α*, *β*, *γ* and *γ′* are set to be 0.5, 0.3, 0.1 and 0.4, respectively.

## 3. Results

This section first discusses the contemporary techniques used in visual place recognition that are compared with our method. Then, we illustrate the experimental results of our method based on the mainstream datasets to outline its performance and its merits for visual place recognition. As mentioned above, the proposed framework can support any kind of CNN structure, therefore in this work, we adopt AlexNet and VGGNet as two examples to illustrate the superiorities over other methods.

### 3.1. Baselines

Some contemporary visual place recognition techniques are compared in this paper:

(1) AMOSNet: Spatial-pyramidal pooling operation is implemented on the conv5 layer of AMOSNet to extract feature vectors. The model is open-sourced [20]. L1-difference is used to measure the distance.
(2) HybridNet: HybridNet and AMOSNet have the same network structure. However, the weights of HybridNet are initialized from CaffeNet. The deployed model parameters of HybridNet are also available [20].
(3) NetVLAD: We have employed the Python implementation of NetVLAD open-sourced in [44]. NetVLAD plug the VLAD layer into the CNN architecture. Given *N* D-dimensional local image feature vectors and *K* cluster centers (“visual words”) as input, the output feature vectors of the VLAD layer are D×K dimensional. The concept of clustering is only used to obtain more global feature vectors in NetVLAD. In contrast to NetVLAD, the clustering method used in this paper is to directly optimize the distance constraint relationships in the Euclidean space. The model selected for evaluation is VGGNet which has been trained in an end-to-end manner on Pittsburgh 250 K dataset [45] with a dictionary size of 64 while performing whitening on the final descriptors.
(4) R-MAC: We have employed the Python implementation for R-MAC [24]. We use conv5_2 of object-centric VGGNet for regions-based features and post-process it with L2 normalization and PCA-whitening [46]. The retrieved R-MACs are mutually matched, followed by aggregation of the mutual regions’ cross-matching scores.
(5) Region-VLAD: We employed conv4 of AlexNet for evaluating the Region-VLAD visual place recognition approach [25]. The employed dictionary contains 256 visual words used for VLAD retrieval. Cosine similarity is subsequently used for descriptor comparison.

### 3.2. Evaluation Datasets

In this experiment, the outdoor dataset New College [47] and the indoor dataset TUM [48] are used. The New College dataset, which contains 1237 image pairs, was collected by the Oxford Mobile Robotics Team. These images were collected by placing a camera on the left and right sides of the mobile platform and acquiring an image every 1.5 m. These images include dynamic objects, and in addition, they were collected on sunny and windy days, which makes the features of images with leaves and shadows unstable. The robot traveled twice in a loop with a total path length of 2 km. When the robot was running around the second loop, it can achieve closed loops.

The TUM dataset is a large dataset containing RGB-D data which is designed to evaluate the vSLAM systems but without ground truths. This dataset contains ground truth poses for key-frames $T_{i},i=1,\ldots ,N$. Therefoe, we can compute the relative distance between the *i*th frame and the *j*th frame according to their poses, which can be shown as below:

(10) $$D_{i,j}=trans(T_{i}^{-1}T_{j})+rot(T_{i}^{-1}T_{j})$$

where the function *trans*(·) and *rot*(·) denotes the translation part and the rotation part of the transform matrix $T_{i}^{-1}T_{j}$. If $D_{i,j}$ is below the given threshold, it means the *i*th frame and the *j*th frame are close from each other, and they are considered to be from the same place.

### 3.3. Results and Analysis

#### 3.3.1. Comparison with Mainstream Methods

**Figure 5:**
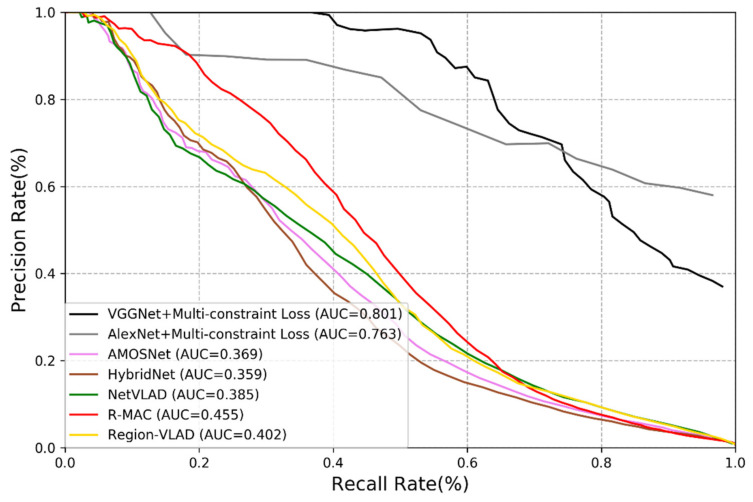
Precision-recall curve of different methods on the New College dataset.

**Figure 6:**
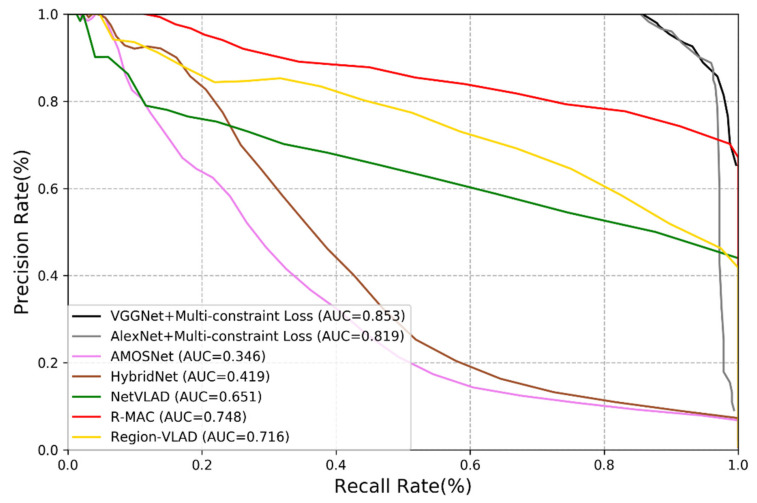
Precision-recall curve of different methods on the TUM dataset.

To verify the performance of the methods used, we calculate the corresponding precision rate and recall rate and plot the Precision-Recall (PR) curves [5]. Figure 5 and Figure 6 show the performance of different methods on the New College dataset and the TUM dataset. We may derive the following remarks:

(1) Among all deep learning-based methods, the performance of AMOSNet, HybridNet and NetVLAD is relatively poor.
(2) Generally, the CNNs trained with multi-constraint loss function exhibit the best performance on both outdoor and indoor datasets. This proves that the multi-constraint loss based deep distance learning is suitable for the visual place recognition and the multi-constraint loss function has great advantages in discriminative feature extraction.
(3) In Figure 5 and Figure 6, the proposed method performs better on the TUM dataset than the New College dataset. It is because images from the TUM dataset are more stable and static and the New College dataset contains more dynamic objects and illumination variations. We may conclude that the proposed method is more suitable for the static indoor environment. This is also valid for NetVLAD, R-MAC and Region-VLAD.
(4) The versatility of the multi-constraint loss is verified in the experiment, i.e., it can support AlexNet, VGGNet and other user-defined networks. In other words, the AMOSNet and HybridNet model can also be combined with the multi-constraint loss for possible further improvement. The influence of the network structure on the performance is not as important as that of the loss function.

#### 3.3.2. Comparison of Multi-Constraint Loss Function and Triplet Loss

**Figure 7:**
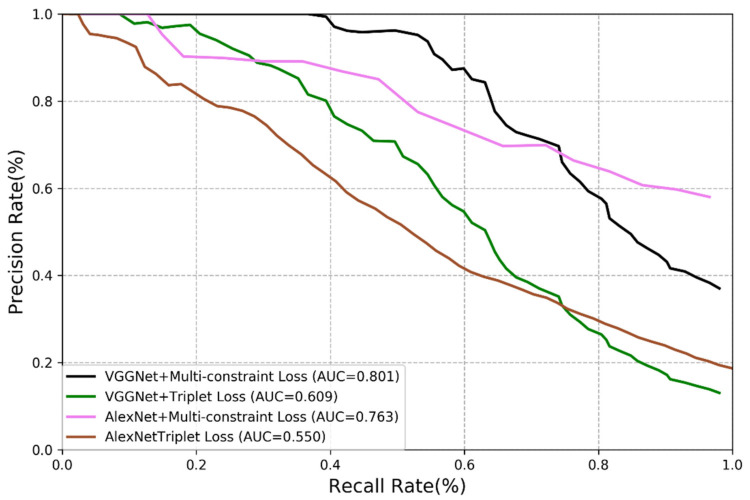
Comparison of multi-constraint loss and triplet loss (New College dataset).

**Figure 8:**
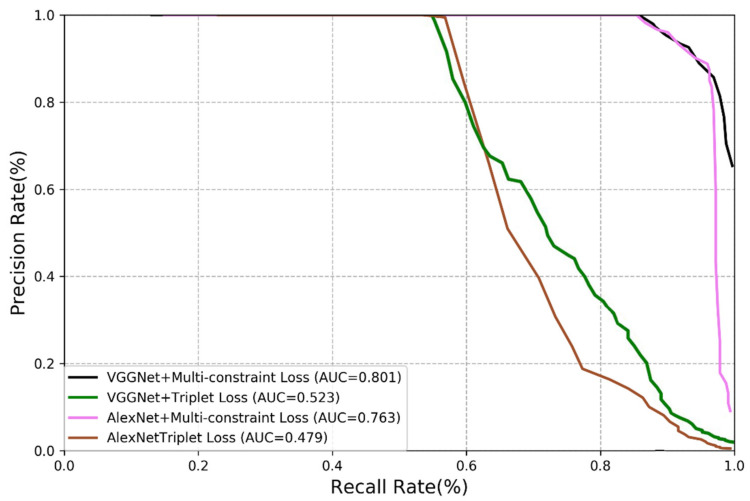
Comparison of multi-constraint loss and triplet loss (TUM dataset).

In this section, we present the experimental results of our method and compare them with those of the triplet loss method [33,34,35]. We carry out comparison on VGGNet and AlexNet using the same experimental steps and network model. The results are shown in Figure 7 and Figure 8.

The results reveal that the loss function is of great significance in visual place recognition. The triplet loss function is originally designed for image classification rather than visual place recognition, it is therefore the improvement on the performance is almost negligible even it is combined with the updated deep learning technology. It is the reason for us to propose the multi-constraint loss function, and it is demonstrated that the multi-constraint loss performs much better than the triplet loss although they worked with the same CNN structures. The features extracted by the multi-constraint based deep distance learning is more essential.

**Figure 9:**
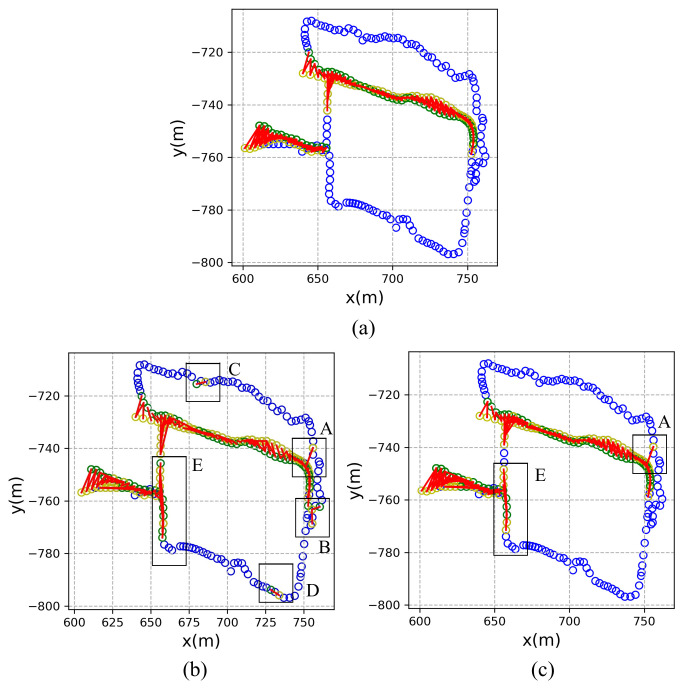
Results visualization of recognized places on the New College dataset. (**a**) The ground-truth trajectory. (**b**) Using triplet loss. (**c**) Using multi-constraint loss.

The effectiveness of the proposed method is further verified in this part by visualization of the results. The New College dataset is taken as an illustrative example. The ground-truth trajectory is shown in Figure 9a. The trajectory of the vehicle is marked as blue circles. Once a place is revisited, the first visiting coordinate is marked as a yellow circle and the revisiting coordinate is marked as a green circle. The recognized image pair is denoted as red lines.

The places recognized by triplet loss and multi-constraint loss are shown in Figure 9b,c, respectively. The results indicate that most of the closed loops can be detected by the proposed method on the New College dataset. It is clearly shown from the visualization that there exist false detected closed loops. In Figure 9b, the results using triplet loss show many false detected closed loops located in area A, B, C, D and E, which indicates unreliable of using the triplet loss in visual place recognition. While in Figure 9c the false detected closed loops can only be found in area A and E.

**Figure 10:**
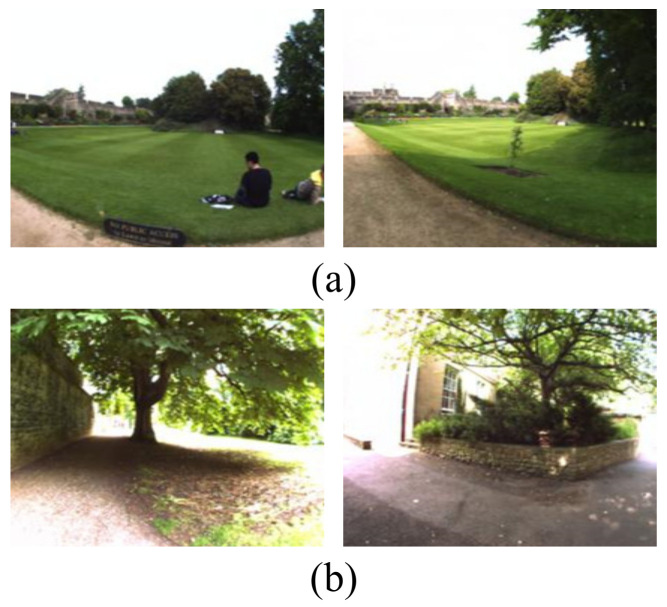
False recognized places (**a**) in area E. (**b**) in area A.

Therefore, we carry out a thorough analysis of the New College dataset to find out the reasons for the false recognized places. The images in area E were taken continuously along a straight road. Figure 10a shows a representative false detected closed loop in area E. These two images are similar in that they contain a large lawn and the same houses in the distance. But they were not shot in the same location. There are many similar situations in area E. It is a hard task for the existing methods to distinguish whether it’s a revisited place or not. Figure 10b shows another example of a false recognized place in area A. These two images contain lots of leaves and shadows, which lead to strong condition variations that both methods are easily confused to have a revisited place.

**Figure 11:**
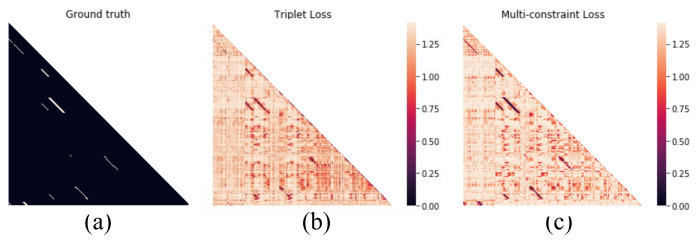
Visualization of the distance matrices. (**a**) The ground truth of the New College dataset. (**b**) The triplet loss. (**c**) The multi-constraint loss.

The proposed method can learn a more discriminative distance preserving embedding. It can be further verified with the visualized distance matrix images. The distance matrices obtained by multi-constraint loss and triplet loss are visualized in Figure 11b,c. These distance matrices are plotted as heatmaps, where bright color means large distance between image pair. From the experimental result, the proposed multi-constraint loss is able to recognize the most revisited places while the triplet loss performs relatively poor. The distance contrast of Figure 11c is more obvious than that of Figure 11b. It means that the proposed method with multi-constraint loss can provide more accurate distance preserving embedding in visual place recognition with complex scenarios because the distances between images from the same place are reduced and those from different places are enlarged.

#### 3.3.3. Comparison of Multi-Constraint Loss Function and Triplet Loss

As previously mentioned, we can extract the low-dimensional feature vector from the fully connected layer of CNN to speed up the visual place recognition process. In the experiment, we set the dimension of the feature vector as 1000, 2500 and 4096, respectively to compare the corresponding performances.

**Figure 12:**
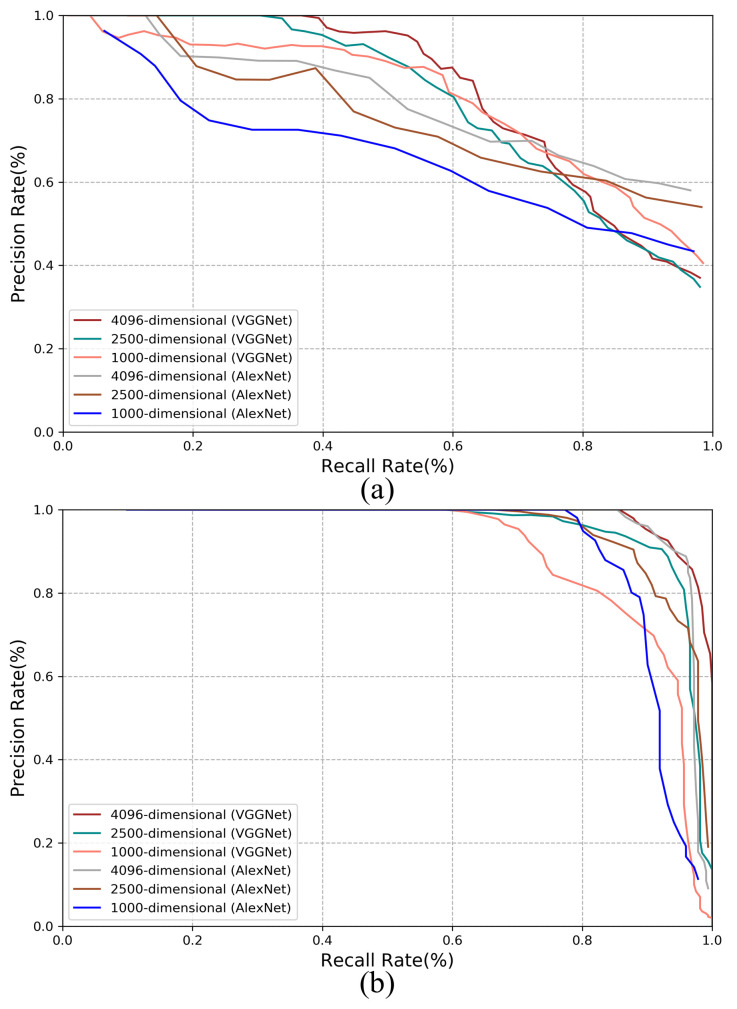
Performance comparison with different dimensions of feature vector (**a**) Results on the New College dataset. (**b**) Results on the TUM dataset.

The results on the New College dataset and the TUM dataset are shown in Figure 12a,b respectively. It is expected that a higher dimension enables better performance. VGGNet can provide slightly better performance than AlexNet because VGGNet has a deeper network structure.

Generally, by using multi-constraint loss, the performance won’t degrade too much when the feature dimension reduces. It indicates that even the vector dimension or the model is compressed into one quarter, the loss of the performance is within 5–10%. Therefore, it is possible to use the proposed method for embedded applications.

#### 3.3.4. Time Performance Comparison

**Figure 13:**
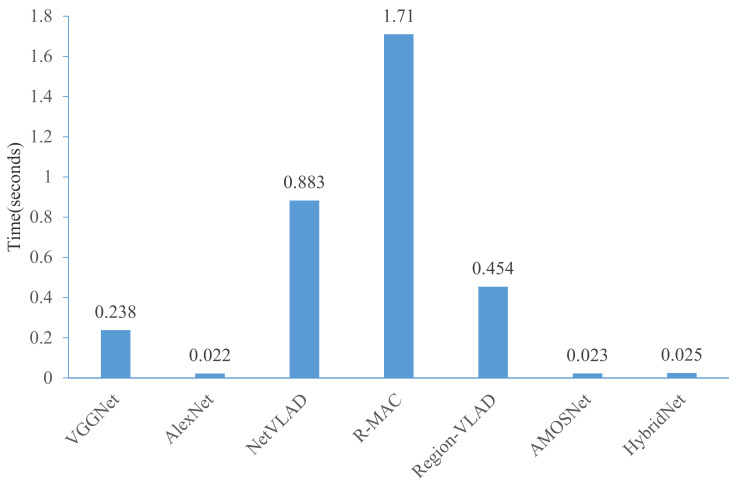
Comparison on feature extraction time.

In this part, the superior of the proposed method for real-time inference is described. First, we compare the average processing time for feature extraction. Given a single query image, the feature extraction time for all visual place recognition techniques is shown in Figure 13. As expected, AlexNet, AMOSNet and HybridNet show the best performance because of the lightweight network structure. The results also indicate that the proposed framework no matter with AlexNet or VGGNet is much more efficient. For AlexNet with multi-constraint loss, the average time reduces to 0.022 s per image pair, almost 77 times faster than that of the R-MAC method. It is noted that in the proposed method, the feature extraction time is only related to the feature extraction network used, and it has nothing to do with the loss function used during training and the dimension of feature vectors. In the future, we may design a faster and more lightweight network model to further reduce the feature extraction time.

**Figure 14:**
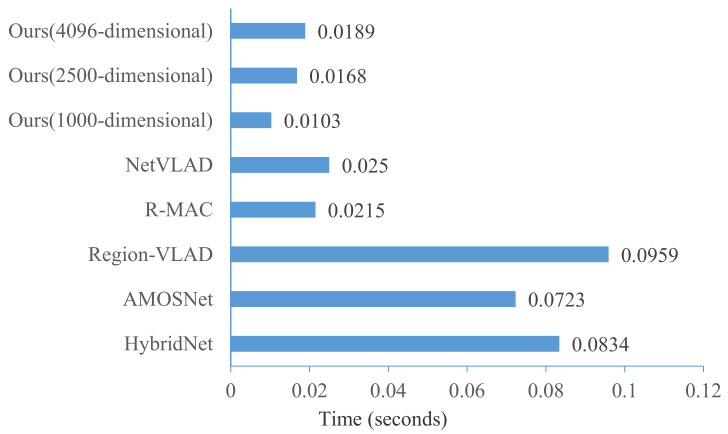
Comparison on similarity metric time.

In visual place recognition, similarity metric time is an important factor that needs to be considered when comparing a query image against a large number of reference images. In this section, we show the time taken to match feature vectors of a query and a reference image in Figure 14. In our method, since we have performed deep distance learning in the Euclidean space, the Euclidean distance can be directly used to metric the similarity of images. Therefore, the similarity metric time is related to the length of the feature vectors. The average similarity metric time in our method with a 4096 dimension vector is around 0.0189 ms, which is fast enough for the real-time inference of large-scale visual place recognition. Thus, the proposed method could be easily plugged on any embed device.

**Figure 15:**
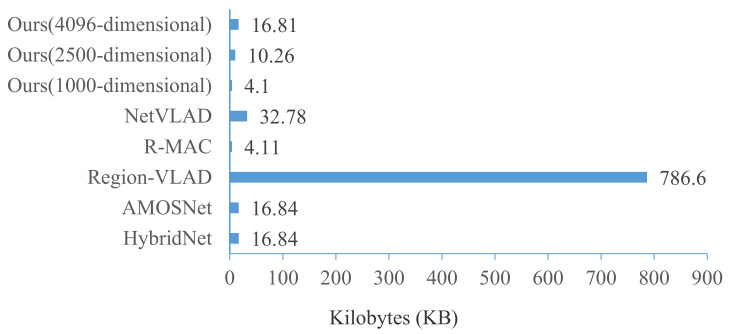
Feature vector sizes of different methods.

Although the performance of visual place recognition can be improved as the feature dimension increases, it may not applicable for real-time inference of visual place recognition tasks. It is of great significance to have a compact feature vector due to the limited resources of the embedded vSLAM systems. The feature vector sizes of different methods are compared in Figure 15. It is interesting to note that Region-VLAD suffers from a significantly higher feature vector size. In the AMOSNet and HybridNet methods, the size of the feature map after feature extraction is (256, 13, 13). In the R-MAC method, the size of the feature vector of each image is 512. In the NetVLAD method, the size of the feature map after feature extraction is 4096. In Figure 15, the feature vector size of our method with 1000-dimensional is the smallest.

## 4. Conclusions

In this paper, we present an effective multi-constraint based deep distance learning framework for visual place recognition. In this model, we can support any kind of CNN network that is trained by the multi-constraint image set to produce more discriminative feature representations that can satisfy the relative distance relationship in visual place recognition. Our learning algorithm ensures the overall computation load mainly depends on the number of training places rather than the number of training images. The results of extensive experiments demonstrate that the proposed method generally outperforms mainstream methods in terms of both effectiveness (precision-recall) and efficiency (runtime). In future research, we plan to integrate our model into various vSLAM systems with complex scenarios. Additionally, the application of our model for visual place recognition in a vSLAM application like urban autonomous driving is of great significance, and the results can be further improved by adding extra false positive rejection methods (i.e., a geometric check).

## Data Availability

The data presented in this study are openly available in [47,48].

## References

[1] Lowry S., Sünderhauf N., Newman P., Leonard J., Cox D., Corke P., Milford M. (2016). Visual place recognition: A survey. IEEE Trans. Rob..

[2] Raúl M., Tardós J. (2017). ORB-SLAM2: An Open-Source SLAM System for Monocular, Stereo, and RGB-D Cameras. IEEE Trans. Robot..

[3] Cadena C., Carlone L., Carrillo H., Latif Y., Scaramuzza D., Neira J., Reid I., Leonard J. (2016). Past, present, and future of simultaneous localization and mapping: Towards the robust-perception age. IEEE Trans. Rob..

[4] Guclu O., Can A. (2019). Fast and Effective Loop Closure Detection to Improve SLAM Performance. J. Intell Robot. Syst..

[5] Zaffar M. (2020). Visual Place Recognition for Autonomous Robots. Master’s Thesis.

[6] Lowe D.R. (2004). Distinctive Image Features from Scale-Invariant Keypoints. Int. J. Comput. Vision..

[7] Bay H., Ess A., Tuytelaars T., Van Goolab L. (2008). Speeded-up robust features (SURF). Comput. Vision Image Underst..

[8] Rublee E., Rabaud V., Konolige K., Bradski G. ORB: An efficient alternative to SIFT or SURF. Proceedings of the IEEE International Conference on Computer Vision.

[9] Dalal N., Triggs B. Histograms of Oriented Gradients for Human Detection. Proceedings of the IEEE Computer Society Conference on Computer Vision and Pattern Recognition.

[10] Sivic J., Zisserman A. Video Google: A Text Retrieval Approach to Object Matching in Videos. Proceedings of the IEEE International Conference on Computer Vision.

[11] Gálvez-López D., Tardos J. (2012). Bags of binary words for fast place recognition in image sequences. IEEE Trans. Rob..

[12] Liu H., Wang R., Shan S., Chen X. Deep Supervised Hashing for Fast Image Retrieval. Proceedings of the IEEE Conference on Computer Vision and Pattern Recognition.

[13] Krizhevsky A., Hinton G.E. ImageNet Classification with Deep Convolutional Neural Networks. Proceedings of the Neural Information Processing Systems.

[14] Babenko A., Slesarev A., Chigorin A., Lempitsky V. Neural Codes for Image Retrieval. Proceedings of the European Conference on Computer Vision.

[15] Chatfield K., Simonyan K., Vedaldi A., Zisserman A. Return of the Devil in the Details: Delving Deep into Convolutional Nets. Proceedings of the British Machine Vision Conference.

[16] Wan J., Wang D., Chu Hong Hoi S., Wu P., Zhu J., Zhang Y., Li J. Deep Learning for Content-Based Image Retrieval: A Comprehensive Study. Proceedings of the 22nd ACM International Conference on Multimedia.

[17] Sünderhauf N., Shirazi S., Dayoub F., Upcroft B. On the performance of ConvNet features for place recognition. Proceedings of the IEEE/RSJ International Conference on Intelligent Robots and Systems.

[18] Deng J., Dong W., Socher R., Li L.-J., Li K., Li F.-F. ImageNet: A Large-Scale Hierarchical Image Database. Proceedings of the IEEE Conference on Computer Vision and Pattern Recognition.

[19] Xia Y., Li J., Qi L., Fan H. Loop closure detection for visual SLAM using PCANet features. Proceedings of the International Joint Conference on Neural Networks.

[20] Chen Z., Jacobson A., Sünderhauf N., Upcroft B., Liu L., Shen C., Reid I., Milford M. Deep learning features at scale for visual place recognition. Proceedings of the 2017 IEEE International Conference on Robotics and Automation.

[21] Sun T., Liu M., Ye H., Yeung D.-Y. (2019). Point-cloud-based place recognition using CNN feature extraction. IEEE Sens. J..

[22] Camara L.G., Gäbert C., Přeučil L. Highly Robust Visual Place Recognition through Spatial Matching of CNN Features. Proceedings of the IEEE International Conference on Robotics and Automation (ICRA).

[23] Karen S., Andrew Z. (2014). Very deep convolutional networks for large-scale image recognition. arXiv.

[24] Tolias G., Sicre R., Jegou H. Particular object retrieval with integral max-pooling of CNN activations. Proceedings of the International Conference on Learning Representations.

[25] Khaliq A., Ehsan S., Milford M., McDonald-Maier K. (2019). A holistic visual place recognition approach using lightweight cnns for significant viewpoint and appearance changes. IEEE Trans. Robot..

[26] Jegou H., Douze M., Schmid C., Perez P. Aggregating local descriptors into a compact image representation. Proceedings of the IEEE Computer Society Conference on Computer Vision and Pattern Recognition.

[27] Zitnick C., Dollar P. Edge boxes: Locating object proposals from edges. Proceedings of the European Conference on Computer Vision.

[28] Sunderhauf N., Shirazi S., Jacobson A., Dayoub F., Pepperell E., Upcroft B., Milford M. Place recognition with ConvNet landmarks: Viewpoint-robust, condition-robust, training-free. Proceedings of the Robotics: Science and Systems.

[29] Milford M.J., Wyeth G.F. SeqSLAM: Visual route-based navigation for sunny summer days and stormy winter nights. Proceedings of the IEEE International Conference on Robotics and Automation (ICRA).

[30] Oishi S., Inoue Y., Miura J., Tanaka S. (2019). SeqSLAM++: View-based robot localization and navigation. Robot. Auton. Syst..

[31] Johns E., Yang G.Z. Feature co-occurrence maps: Appearance-based localisation throughout the day. Proceedings of the IEEE International Conference on Robotics and Automation (ICRA).

[32] Ho K.L., Newman P.M. (2007). Detecting loop closure with scene sequences. Int. J. Comput. Vision..

[33] Gu J., Wang Z., Kuen J., Ma L., Shahroudy A., Shuai B., Liu T., Wang X., Wang G., Cai J. (2018). Recent advances in convolutional neural networks. Pattern Recognit..

[34] Hermans A., Beyer L., Leibe B. (2017). In defense of the triplet loss for person re-identification. arXiv.

[35] Liu H., Feng J., Qi M., Jiang J. (2017). End-to-end comparative attention networks for person re-identification. IEEE Trans. Image Process..

[36] Xie S., Pan C., Peng Y., Liu K., Ying S. (2020). Large-Scale Place Recognition Based on Camera-LiDAR Fused Descriptor. Sensors.

[37] Martini D., Gadd M., Newman P. (2020). kRadar++: Coarse-to-Fine FMCW Scanning Radar Localisation. Sensors.

[38] Săftescu Ş., Gadd M., Martini D., Barnes D., Newman P. (2020). Kidnapped Radar: Topological Radar Localisation using Rotationally-Invariant Metric Learning. arXiv.

[39] Gadd M., Martini D.D., Newman P. Look around You: Sequence-based Radar Place Recognition with Learned Rotational Invariance. Proceedings of the IEEE/ION Position, Location and Navigation Symposium (PLANS).

[40] Zheng L., Yang Y., Tian Q. (2018). SIFT meets CNN: A decade survey of instance retrieval. IEEE Trans. Pattern. Anal. Mach. Intell..

[41] Azizpour H., Razavian A., Sullivan J., Maki A. (2016). Factors of Transferability for a Generic ConvNet Representation. IEEE Trans. Pattern. Anal. Mach. Intell..

[42] Zheng L., Zhao Y., Wang S., Wang J., Tian Q. (2016). Good practice in CNN feature transfer. arXiv.

[43] Jin S., Gao Y., Chen L. (2020). Improved Deep Distance Learning for Visual Loop Closure Detection in Smart City. Peer-to-Peer Netw. Appl..

[44] Cieslewski T., Choudhary S., Scaramuzza D. Data-efficient decentralized visual SLAM. Proceedings of the 2018 IEEE International Conference on Robotics and Automation.

[45] Arandjelovi R., Gronat P., Torii A., Pajdla T., Sivic J. (2018). NetVLAD: CNN Architecture for Weakly Supervised Place Recognition. IEEE Trans. Pattern Anal. Mach. Intell..

[46] Jégou H., Chum O. Negative Evidences and Co-occurences in Image Retrieval: The Benefit of PCA and Whitening. Proceedings of the European Conference on Computer Vision.

[47] Cummins M., Newman P. (2008). FAB-MAP: Probabilistic localization and mapping in the space of appearance. Int. J. Rob. Res..

[48] Sturm J., Engelhard N., Endres F., Burgard W., Cremers D. A benchmark for the evaluation of RGB-D SLAM systems. Proceedings of the IEEE/RSJ International Conference on Intelligent Robots and Systems.

